# From arid deserts to mesic meadows: divergent pathways regulating microbial respiration under grassland enclosure

**DOI:** 10.3389/fmicb.2025.1594877

**Published:** 2025-10-27

**Authors:** Asitaiken Julihaiti, Dong Yiqiang, Zhou Shijie, Nie Tingting, Jiang Anjing, An Shazhou

**Affiliations:** ^1^School of Grassland, Xinjiang Agricultural University, Urumqi, China; ^2^Key Laboratory of Grassland Resources and Ecology Autonomous Region, Urumqi, China; ^3^Key Laboratory of Grassland Resources and Ecology, Ministry of Education, Urumqi, China

**Keywords:** enclosure, grassland types, microbial assembly process, microbial respiration, Partial Least Squares Path Modeling (PLS-PM)

## Abstract

**Introduction:**

As a pivotal restoration strategy for alleviating grassland degradation, long-term enclosure practices effectively eliminate livestock disturbances while facilitating ecosystem self-recovery. Understanding the dynamics of soil microbial respiration under enclosure management is crucial, as it provides a scientific foundation for optimizing grassland utilization and contributes to global research on the terrestrial carbon cycle.

**Methods:**

We conducted a comparative study across three distinct enclosed grassland ecosystems in Xinjiang, China: temperate desert, temperate steppe, and mountain meadow. Through analyzing microbial community structure, diversity, assembly processes, and respiration rates between 9-year enclosed and grazed areas, we identified key ecological shifts.

**Results:**

Three key advancements emerged: (1) Enclosure implementation led to a marked improvement in soil resource availability, triggering microbial community shifts from oligotrophic to eutrophic states with substantial biodiversity increases (bacterial diversity: 2.2–14%; fungal diversity: 12.4–27.2%); (2) Divergent assembly mechanisms were observed where surface soil bacterial communities (0–5 cm depth) transitioned from 55.6 to 100% deterministic processes, directly contrasting with fungal communities that shifted from 11.1 to 55.6% stochastic dominance; (3) Partial Least Squares Path Modeling (PLS-PM) revealed distinct ecosystem-specific regulatory mechanisms underpinning reduced microbial respiration.

**Discussion:**

The PLS-PM analysis detailed these distinct mechanisms: soil property-induced microbial metabolic trade-offs enhanced carbon use efficiency in temperate desert (*R*^2^ = 0.951), plant-mediated microbial assembly processes promoted efficient carbon cycling in temperate steppe (*R*^2^ = 0.455), and plant-driven suppression of microbial biomass dominated respiratory reduction in mountain meadow (*R*^2^ = 0.883). The research establishes that enclosure achieves carbon sequestration through divergent pathways across ecosystems, providing critical insights for optimizing grassland management strategies and enhancing climate change mitigation efforts.

## 1 Introduction

As fundamental components of grassland ecosystems, soil microorganisms play a key role in decomposing plant biomass and organic residues, while simultaneously driving biogeochemical cycles and energy fluxes within these ecosystems. Their critical role in maintaining ecological equilibrium and serving as sensitive bioindicators of environmental change has been well documented (Huang et al., 2009). Contemporary microbial ecology research has undergone a paradigm shift toward investigating microbial community assembly mechanisms (Jiao et al., 2020; Stegen et al., 2012; Stegen et al., 2013; Nemergut et al., 2013; Dini-Andreote et al., 2015). Current scientific consensus holds that microbial assemblage dynamics are governed by an interplay of deterministic and stochastic processes (Stegen et al., 2012; Zhang et al., 2022; Wu et al., 2022; Dumbrell et al., 2010). Deterministic processes encompass both abiotic factors (environmental filtering and selection pressures) and biotic interactions (including microbial antagonism and mutualism), which collectively determine species survival, extinction probabilities, and relative abundance distributions within communities (Stegen et al., 2012; Vellend, 2010). Conversely, stochastic processes involve unpredictable disturbances, probabilistic dispersal limitations, and random birth-death events that occur independently of environmental gradients (Dini-Andreote et al., 2015; Chase and Myers, 2011). This theoretical framework has been extensively applied to elucidate microbial community formation (Fukami, 2015; Ernakovich et al., 2022) and provides novel insights into microbial responses to environmental perturbations (Nemergut et al., 2013; Dini-Andreote et al., 2015). Nevertheless, significant scientific debate persists regarding the relative contributions of deterministic versus stochastic processes in shaping microbial community structure and function (Nemergut et al., 2013; Ernakovich et al., 2022; Knelman and Nemergut, 2014; Tucker et al., 2016).

As the most biologically active components within soil ecosystems, soil microorganisms drive biogeochemical processes through their metabolic activities. Microbial respiration—the terminal process of organic carbon decomposition—serves as the primary conduit for soil-to-atmosphere CO₂ release, regulated by abiotic factors (temperature, moisture, soil properties) and ecosystem-specific variables (vegetation type, land management, anthropogenic disturbances), generating predictable and stochastic flux patterns (Raich and Schlesinger, 1992). Plant diversity enhances microbial biomass via substrate diversity and nutrient availability, establishing a direct link between floristic richness and elevated respiration rates (Lange et al., 2015; Eisenhauer et al., 2013; Khlifa et al., 2017; Ma and Chen, 2018; Tilman et al., 2001). These biome-specific regulatory mechanisms—though requiring empirical validation in carbon flux studies—provide a conceptual framework to interpret divergent respiration patterns across grasslands: deterministic assembly under resource enrichment (e.g., enclosure-induced nutrient availability) selects copiotrophic bacteria with high metabolic activity, accelerating labile carbon mineralization, while stochastic assembly in fungal communities promotes decomposition of complex substrates (e.g., lignin) and carbon stabilization through functional niche complementarity (Ernakovich et al., 2022; Knelman and Nemergut, 2014).

Grazing, as the most prevalent and cost-effective grassland management practice globally, often triggers vegetation degradation under unsustainable intensities, compromising multifunctionality through diminished ecological services (e.g., carbon sequestration, soil stabilization) and pastoral productivity (Dong, 2016). In response, grassland enclosures have been widely adopted to restore degraded ecosystems by eliminating herbivore pressure, thereby facilitating natural regeneration of vegetation and soil biota (Chen et al., 2018; Ju et al., 2024). Contemporary studies emphasize that enclosure-induced shifts in soil properties and plant communities restructure microbial assembly processes (deterministic vs. stochastic), which subsequently modulate microbial respiration and ecosystem carbon cycling (Hu et al., 2024). Nevertheless, critical gaps persist in understanding: (a) how soil–plant-microbe interactions collectively regulate respiration pathways; (b) the legacy effects of historical grazing on microbial functional redundancy; and (c) the metabolic constraints governing carbon flux trajectories during recovery (Wu et al., 2017).

Building upon this theoretical framework, our study conducted a comparative analysis of three grassland ecosystems subjected to 9-year grazing exclusion along the northern slope of the Tianshan Mountains: temperate desert, temperate steppe, and mountain meadow. Through comprehensive characterization of soil microbial community structure, diversity profiles, and respiratory activity across enclosed and grazed plots, we hypothesize that: (1) Bacteria-fungi assembly dichotomy: Functional divergence drives bacterial communities toward deterministic dominance via environmental filtering under resource enrichment (after enclosure), while fungal communities exhibit stochastic assembly through dispersal strategies and ecological drift in enriched environments (after enclosure); and (2) grazing exclusion induces distinct biome-specific pathways regulating soil microbial respiration: (i) in the fragile temperate desert, direct abiotic forcing via enclosure-triggered soil modifications will rapidly reconfigure nutrient dynamics to mediate respiration; (ii) in the semi-arid temperate steppe, vegetation recovery (e.g., increased dominance but declined diversity) will establish biotic control by restructuring microbial communities and assembly processes; and (iii) in the mesic mountain meadow, plant-driven suppression of microbial biomass will override other factors to ultimately limit respiratory carbon loss. Furthermore, we predict that these divergent pathways will hierarchically enhance ecosystem carbon retention under enclosure management. This investigation aims to elucidate how enclosure-induced changes in microbial assembly regulate soil microbial respiration, and thereby to establish predictive microbial assembly-ecosystem recovery linkages, identify biome-specific carbon cycling responses, and provide microbial-mediated management strategies for degraded grasslands.

## 2 Materials and methods

### 2.1 Study area

**Figure 1:**
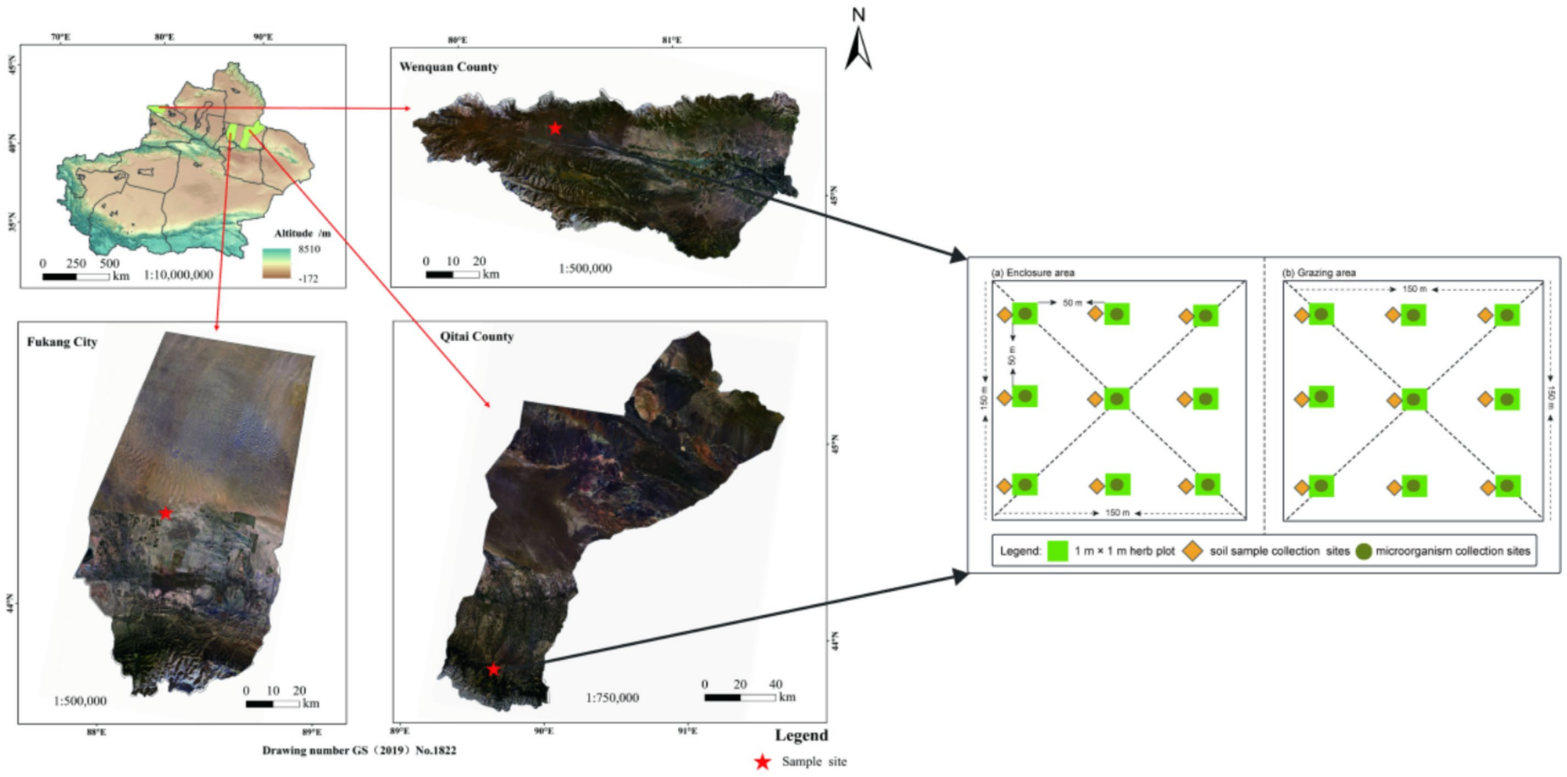
Digital elevation map showing the locations of the three sampled sites in the Tianshan Mountains.

The research area encompasses Wenquan County of the Boltala Mongolian Autonomous Prefecture in the western region, as well as Fukang City and Qitai County, situated in the Changji Hui Autonomous Prefecture, to the east of the northern slope of the Tianshan Mountain. The fundamental details of the sample points are outlined in Figure 1 and Supplementary Table S1. Specifically, within the study area of Fukang City, the grassland type is classified as temperate desert, featuring sierozem soil and a sandy soil texture. The dominant flora comprises *Haloxylon ammodendron* and *Seriphidium santolinum*, with *Kali collinum* as an associated species. On the other hand, the grassland type in the Wenquan County research area is temperate steppe, characterized by chestnut soil and a loam soil texture. The primary flora includes *Stipa capillata*, *Festuca ovina*, and *Artemisia frigida*, with *Aster altaicus* as an associated species. Furthermore, the Qitai County research area is classified as mountain meadow, with chernozem soil and a loam soil texture. The dominant plant species is *Alchemilla tianschanica*, while the main associated species encompass *Bromus inermis*, *Polygonum viviparum*, *Trifolium lupinaster*, *Carex liparocarpos*, *Astragalus membranaceus*, and *Geranium carolinianum*, among others.

### 2.2 Experiment design

The test sites were strategically positioned within the designated national fixed monitoring sites, each encompassing an area of approximately 3 × 10^4^ m^2^. Two treatments are set in temperate desert (TD), temperate steppe (TS), and mountain meadow (MM), respectively: grazing exclusion plots (GE) and freely grazing plots (FG). Notably, all these exclusion plots have been enclosed since 2012 and remained enclosed for a duration of 9 years by the sampling year of 2021. The overall stocking rate for the three grassland sites ranged from 0.90 to 4.50 sheep units per hectare (hm^2^). The grazing intensities at these three sites fell within the category of moderate grazing in the local context, aligning with the carrying capacity of livestock in the area. Before enclosure, the enclosed areas exhibited comparable vegetation composition, community characteristics, and landforms to the control grazing areas.

### 2.3 Plant and soil sampling and determination

Vegetation surveys were conducted during peak biomass season (July 2021) across enclosure and grazed sites. Three 50-m spaced transects per treatment contained three 1 × 1 m quadrats (50-m intervals; 54 quadrats total). Plant species composition was recorded, with coverage (%) assessed by the point-intercept method, height (cm) by tape measure, density (plants·m^−2^) by direct counting, and above-ground biomass (AGB) by harvesting, oven-drying (105 °C/30 min → 80 °C/24 h), and weighing. Below-ground biomass (BGB) was determined from intact soil blocks (10 × 20 cm) after washing and drying [methods follow Liu et al. (2022)].

Soil sampling occurred concurrently within vegetation quadrats. Stratified samples (0–5 and 5–10 cm depths) were homogenized per layer, with subsamples stored at 4 °C (microbial assays) or air-dried (physicochemical analysis). Standard protocols quantified: (1) pH (soil:water = 5:1); (2) SOC (dichromate oxidation); (3) N/P fractions (Kjeldahl N; Mo-Sb colorimetric P); and (4) Microbial biomass (fumigation-extraction for MBC/MBN/MBP) (Liu et al., 2022) (Full analytical details in Appendix A). All datasets are original. Plant/soil data will be analyzed in a companion study currently in preparation, with no content overlap between manuscripts.

### 2.4 Soil microbial sampling and determination

In July 2021, the soil microorganism sample collection was carried out, and three typical samples were set up in each of the treatment area and the control area, with a sample line of 50 m, and the sample of three 1 m × 1 m on each sample line, and the sample space was about 50 m (Figure 1). First, the soil microorganism samples were collected from the soil 0–5 cm and the 5–10 cm layer, and each sample line was mixed evenly and then put in the car refrigerator (−20 °C) in a sealed bag and brought back to the laboratory. The study of soil microorganism samples by high-pass sequencing analysis of community structure and diversity, the research steps are: (1) to extract the total DNA in the sample using the Hongkey group DNA extraction kit. (2) the V3 ~ V4 variable zone sequence of the bacterium 16 s rRNA gene is the ITS1-1f zone of its rRNA gene, with the 338f-806r of the barcode sequence (the fungal derivative is the ITS1-1f-fdon ITS1-1f-r), and the PCR amplification is used to obtain the PCR product (Ju et al., 2024; Caporaso et al., 2011). (3) After the PCR product was built by quantitative and library, the Illumina MiSeq PE 300 platform was used to sequence the high pass, and to obtain the information of the base sequence of bacterial and fungal variable areas. (4) using the QIIME2.0 package, the OTU (taxonomic operational unit) cluster, the OTU represents the sequence and the SILVA database (Fungi for UNITE database) for the comparison analysis, and obtain the OTU corresponding classification unit and its corresponding quantity of abundance information (Jiang et al., 2022). (5) through the analysis of the microbial sequence, the abundance information of the microbiogenic cloud platform of the micro group, the discovery of *α* diversity, and so on. The analysis of the microbiological sequencing of the study was completed in the city of Shenzhen Microleague Technology Group Co., LTD (Jiang et al., 2022). The soil microbial respiration (SMR) was measured using an automatic soil aerobic culture system (He et al., 2013; Zhang et al., 2025).

The raw microbial sequence data from this study have been deposited in the Figshare repository and are publicly available under the DOI https://doi.org/10.6084/m9.figshare.30024817.v1 and at NCBI BioProject, accession PRJNA1338829 (Figshare permanent link: https://figshare.com/articles/figure/microbial_raw_sequence_data/30024817). These data are publicly available as of the date of publication.

### 2.5 Data statistics and analysis

Primary data preprocessing was performed using Microsoft Excel 2020 (Microsoft Corp.). Microbial α-diversity indices and soil microbial respiration metrics across grassland types were statistically analyzed through IBM SPSS Statistics 25.0 (IBM Corp.) employing a hierarchical analytical framework: (1) Before parametric tests, homogeneity of variances was assessed using Levene’s test (α = 0.05). For variables violating homogeneity (*p* < 0.05), Welch’s ANOVA or non-parametric Kruskal-Wallis tests were applied. (2) One-way ANOVA with post-hoc Tukey tests for inter-biome comparisons; (3) Multifactorial ANOVA to disentangle enclosure effects from ecosystem-type interactions; and (4) Independent-sample *t*-tests for paired enclosure vs. grazed plot analyzes. Microbial community assembly processes were quantified using the *β*-nearest taxon index (βNTI) and normalized stochasticity ratio (NST) computed through the “picante” (v1.8.2) and “NST” (v3.0.3) packages in R 4.2.1 (R Core Team). Deterministic versus stochastic dominance was assessed using established ecological thresholds (βNTI > 2 or βNTI < −2 indicates deterministic assembly; −2 < βNTI < 2 reflects stochastic dominance).

Partial Least Squares Path Modeling (PLS-PM) was performed using the plspm package (v0.4.9) in R 4.2.1 to elucidate the direct and indirect effects of environmental covariates on microbial respiration. This method was selected for its robustness with small sample sizes and its suitability for exploratory research and complex model structures. The analysis was designed to: (1) Decouple the direct and indirect effects along hypothesized pathways linking enclosure measures, soil properties (e.g., pH, SOC, TN), plant characteristics (e.g., above-ground biomass, below-ground biomass, richness), microbial properties (e.g., microbial biomass carbon, nitrogen), and microbial respiration; and (2) Test the proposed ecological mechanisms governing respiration dynamics in each grassland type. The model’s predictive accuracy and explanatory power were evaluated using the coefficient of determination (*R*^2^) for endogenous latent variables. The overall model quality was assessed by the Goodness-of-Fit (GoF) index. The significance of path coefficients was determined through a bootstrap validation procedure with 5,000 iterations to generate robust standard errors and confidence intervals.

All statistical graphics were programmatically generated using “ggplot2” (v3.4.2) in R 4.2.1, with subsequent vector refinement and compositional optimization performed in Adobe Illustrator 2021 (Adobe Inc.). Data are presented as mean ± standard error (SE).

## 3 Results

### 3.1 Effects of enclosure on microbial community structure of different grassland types

**Figure 2:**
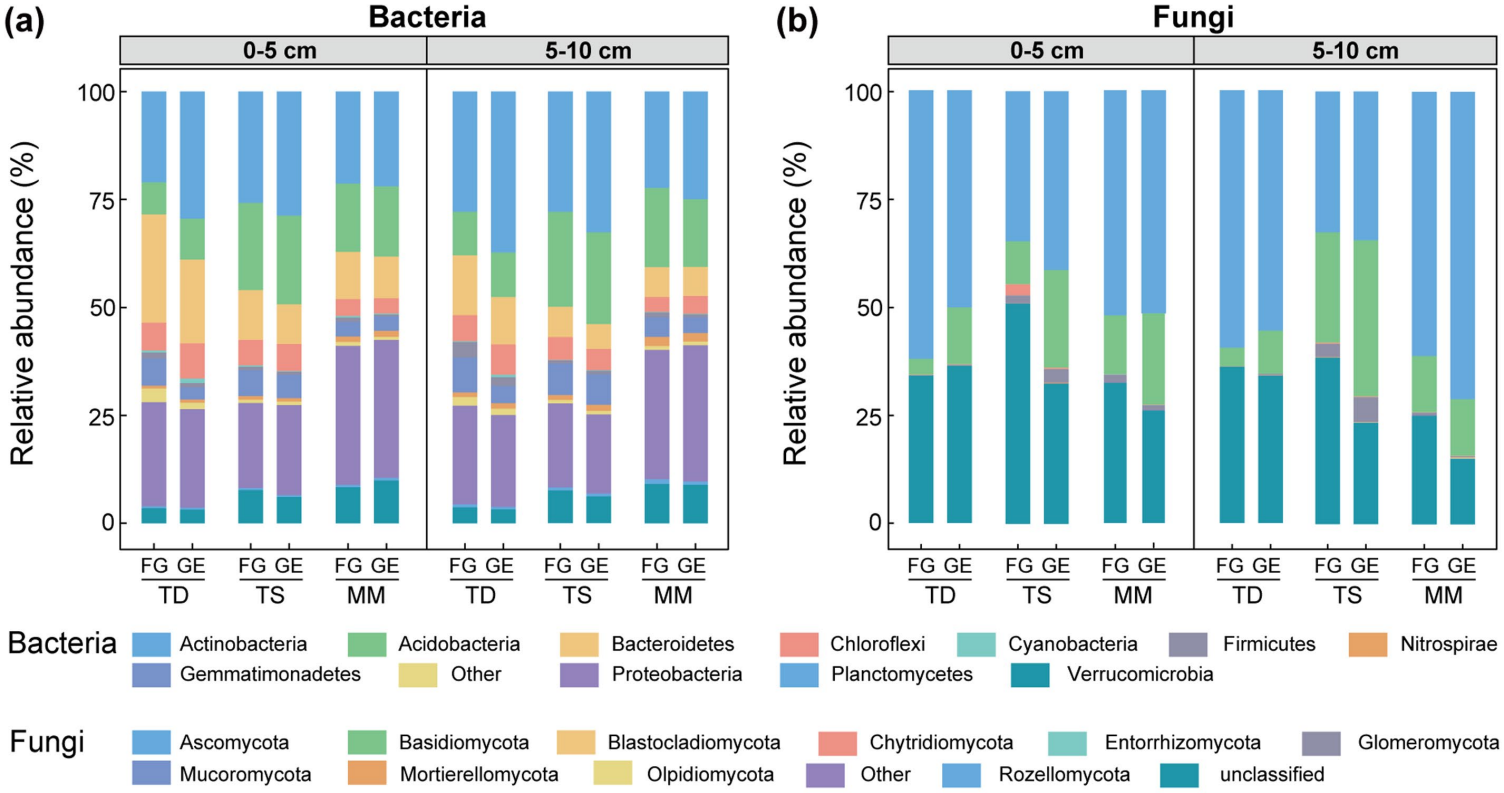
Effects of enclosure on microbial community structure at phylum level of different grassland types. **(a)** Bacteria relative abundance, **(b)** fungi relative abundance. TD (temperate desert), TS (temperate steppe), MM (mountain meadow), GE (grazing exclusion plots), FG (freely grazing plots).

Bacterial phyla (Figure 2a): Actinobacteria, Proteobacteria, Acidobacteria, and Bacteroidetes dominated all grassland types and soil layers. After enclosure, Actinobacteria and Acidobacteria increased by 0.66–8.44% and 0.41–2.03% respectively, in the 0–5 cm layer of all grasslands, while Proteobacteria showed a 1.23% increase exclusively in a temperate steppe. Conversely, Bacteroidetes, Gemmatimonadetes, and Firmicutes decreased by 1.29–5.64%, 0.04–3.50%, and 0.05–0.53% in this layer. In the 5–10 cm layer, Actinobacteria increased by 2.60–9.41%, whereas Bacteroidetes, Verrucomicrobia, and Gemmatimonadetes decreased by 0.17–2.86%, 0.19–1.37%, and 0.28–4.09%, respectively.

Fungal phyla (Figure 2b): Ascomycota and Basidiomycota dominated all grasslands and soil layers, with other phyla (excluding Glomeromycota) accounting for <1% relative abundance. After enclosure, Basidiomycota increased by 7.54–12.64% in the 0–5 cm layer of all grasslands, while Ascomycota showed a 6.62% increase exclusively in a temperate steppe. In the 5–10 cm layer, Basidiomycota increased by 0.13–10.58% across all grasslands, while Ascomycota increased by 1.82–9.95% in temperate steppe and mountain meadow.

### 3.2 Effects of enclosure on microbial diversity in different grassland types

**Figure 3:**
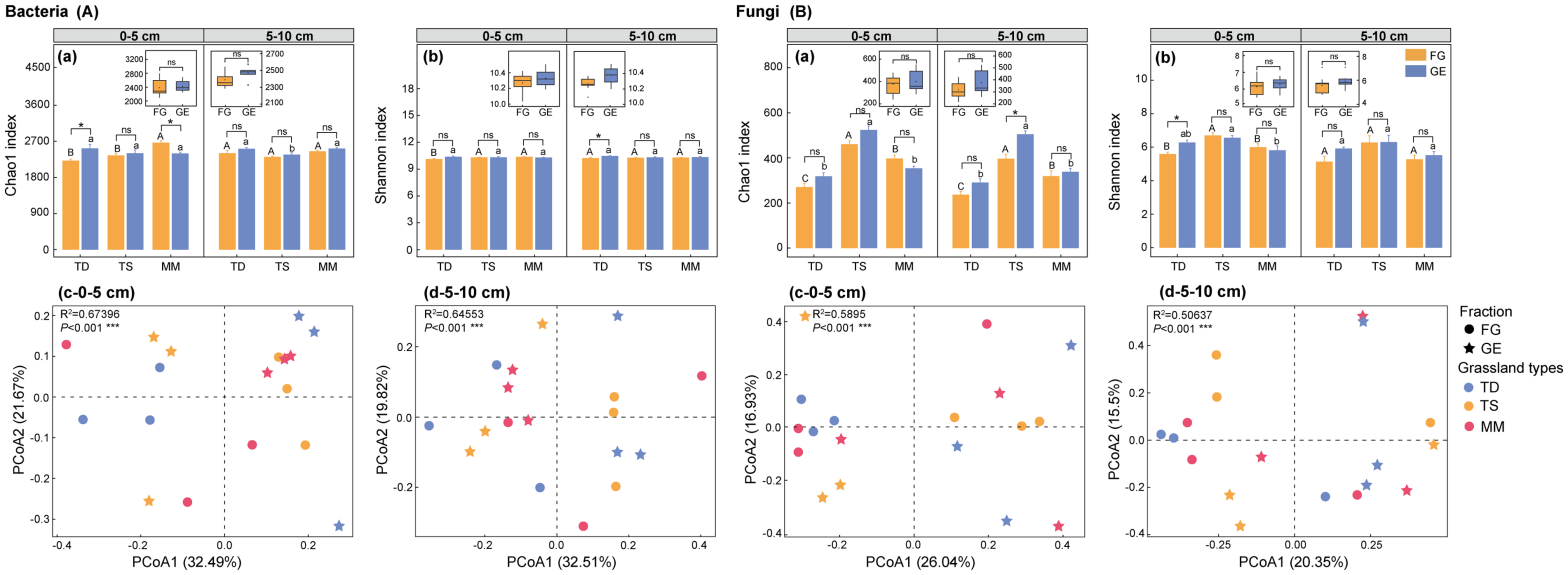
Effects of enclosure on microbial community diversity of different grassland types. **(A)** bacteria diversity index, **(B)** fungi diversity index, **(a)** bacterial and fungal Chao1 index, **(b)** bacterial and fungal Shannon index, **(c)** bacterial and fungal 0–5 cm soil layer microbial *β* diversity, **(d)** bacterial and fungal 5–10 cm soil layer microbial *β* diversity. All data points and error bars in histograms represent mean ± standard error. “*,” “**” and “***” respectively indicated significant (*p* < 0.05), extremely significant (*p* < 0.01) and (*p* < 0.001) differences among different treatments of the same grassland type as determined by independent-sample *T* tests, while “ns” indicated no significant (*p* > 0.05) differences; Capital letters: Significant differences between grazing areas of different grassland types (*p* < 0.05) as determined by one-way ANOVA followed by Duncan’s multiple range test; Lowercase letters: Differences between enclosure areas of different grassland types (*p* < 0.05) analyzed with the same method, where shared letters indicate no significant differences (*p* > 0.05); TD (temperate desert), TS (temperate steppe), MM (mountain meadow), GE (grazing exclusion plots), FG (freely grazing plots); Box plots: Combined analysis of enclosure vs. grazing effects across all three grassland types as determined by one-way ANOVA followed by Duncan’s multiple range test.

Enclosure-induced divergent bacterial alpha-diversity responses across grassland ecosystems and soil depths. After enclosure, in the 0–5 cm layer, the Chao1 index increased by 14.0% (*p* < 0.05) in the temperate desert but decreased by 10.0% (*p* < 0.05) in the mountain meadow, whereas no significant changes occurred in the temperate steppe [Figure 3A(a)]. At 5–10 cm depth, after enclosure, only the temperate desert exhibited a response, with a 2.2% increase in Shannon index [*p* < 0.05, Figure 3A(b)]. Beta diversity analysis (Bray-Curtis PCoA) demonstrated strong grassland-type-driven segregation (*p* < 0.001), with bacterial communities of temperate desert and steppe separated along PCoA axis 1 (32.49–32.51% variance explained), independent of soil depth [Figures 3A(c,d)].

Fungal communities displayed contrasting layer-specific enclosure effects. After enclosure, in surface soil (0–5 cm), the Shannon index increased by 12.4% (*p* < 0.05) in the temperate desert, while subsurface soil (5–10 cm) showed a 27.2% Chao1 index rise (*p* < 0.05) in the temperate steppe [Figures 3B(a,b)]. Principal coordinates analysis (PCoA) demonstrated distinct stratification patterns: in 0–5 cm soil layer, temperate desert, and steppe ecosystems exhibited primary fungal community segregation along PCoA axis 1 [26.04% variance, Figure 3B(c)], and in 5–10 cm soil layer, temperate steppe communities showed predominant separation along PCoA axis 2 [15.5% variance, Figure 3B(d)].

### 3.3 Effects of enclosure on microbial assembly process of different grassland types

**Figure 4:**
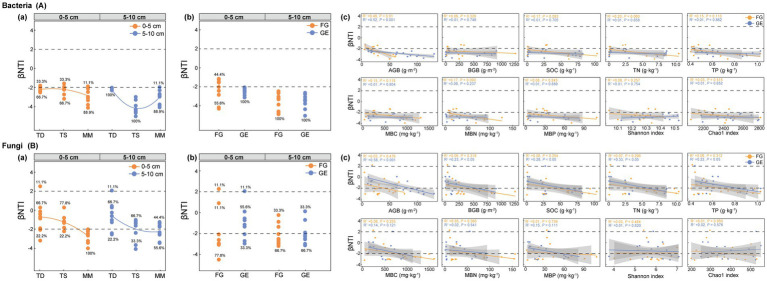
Effects of enclosure on microbial assembly in different grassland types. **(A)** Bacteria assembly process, **(B)** fungi assembly process, **(a)** microbial assembly process in different grassland types, **(b)** microbial assembly process in grazing and enclosure area. **(c)** Trends analyzed by linear regression; AGB (above-ground biomass), BGB (below-ground biomass), SOC (soil organic carbon), TN (total nitrogen), TP (total phosphorus), MBC (microbial biomass carbon), MBN (microbial biomass nitrogen), MBP (microbial biomass phosphorus), FG (freely grazing), GE (grazing exclusion).

As shown in Figure 4, after grassland enclosure, the assembly of soil bacterial communities (βNTI) in both soil layers (0–5 cm and 5–10 cm) across all three grassland types shifted to complete dominance (100%) by deterministic processes [Figure 4A(a)]. Critically, in the 0–5 cm layer before enclosure, bacterial assembly exhibited a mixed process (55.6% deterministic + 44.4% stochastic), which transitioned entirely to deterministic processes (100%) following enclosure [Figure 4A(b)].

For fungal communities under enclosure conditions, stochastic processes dominated assembly in both soil layers of the temperate desert and the temperate steppe, whereas deterministic processes prevailed in the mountain meadow [Figure 4B(a)]. In the 0–5 cm layer, pre-enclosure fungal assembly was predominantly deterministic (88.9%), but shifted to stochastic dominance (55.6%) post-enclosure [Figure 4B(b)]. This directional reversal in the topsoil, from mixed to deterministic for bacteria versus deterministic to stochastic for fungi, highlights an opposing response to enclosure management. These assembly shifts provide the mechanistic basis for the respiration patterns analyzed in Section 3.5.

By analyzing the linear relationships between plant properties, soil characteristics, microbial diversity, and soil microbial assembly processes, our results demonstrate that grassland enclosure management shifted microbial community assembly from predominantly stochastic toward deterministic processes; specifically, for bacterial βNTI, aboveground biomass (AGB) was the dominant driver [*R*^2^ = 0.52, *p* < 0.001; Figure 4A(c)], while for fungal βNTI, AGB showed the strongest correlation (*R*^2^ = 0.58, *p* < 0.001), with significant but weaker associations observed for belowground biomass (BGB: *R*^2^ = 0.23, *p* < 0.05), soil organic carbon (SOC: *R*^2^ = 0.28, *p* < 0.05), total nitrogen (TN: *R*^2^ = 0.33, *p* < 0.05), and total phosphorus (TP: *R*^2^ = 0.22, *p* < 0.05) [Figure 4B(c)].

### 3.4 Effects of enclosure on soil microbial respiration of different grassland types

**Figure 5:**
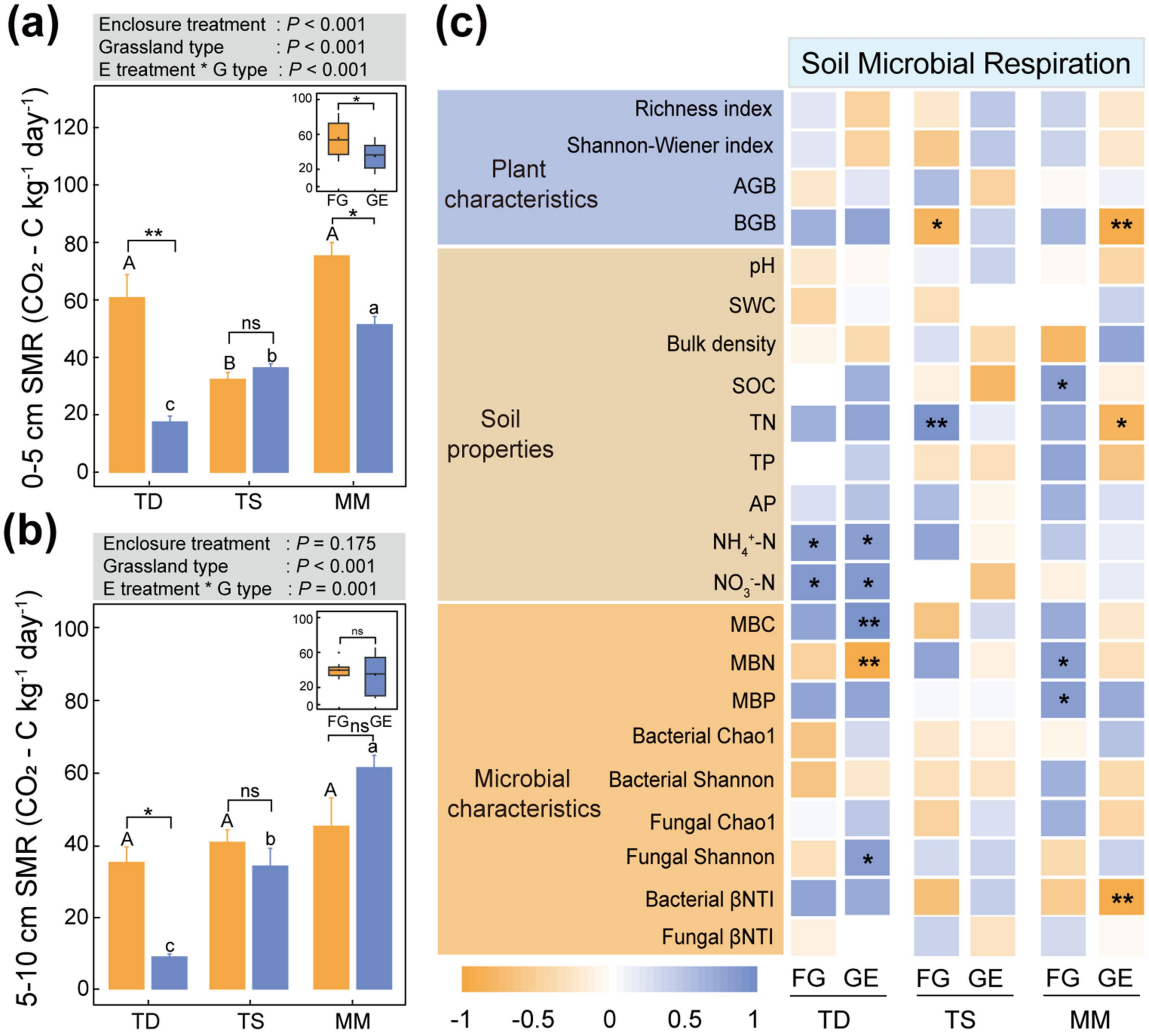
Effects of enclosure on soil microbial respiration of different grassland types. **(a)** 0–5 cm soil microbial respiration, **(b)** 5–10 cm soil microbial respiration. All data points and error bars in histograms represent mean ± standard error. “*,” “**” and “***” respectively indicated significant (*p* < 0.05), extremely significant (*p* < 0.01) and (*p* < 0.001) differences among different treatments of the same grassland type as determined by independent-sample *T* tests, while “ns” indicated no significant (*p* > 0.05) differences; Capital letters: Significant differences between grazing areas of different grassland types (*p* < 0.05) as determined by one-way ANOVA followed by Duncan’s multiple range test; Lowercase letters: Differences between enclosure areas of different grassland types (*p* < 0.05) analyzed with the same method, where shared letters indicate no significant differences (*p* > 0.05); TD (temperate desert), TS (temperate steppe), MM (mountain meadow), GE (grazing exclusion plots), FG (freely grazing plots); Gray box above histograms: Results of two-way ANOVA for the displayed metric; Box plots: Combined analysis of enclosure vs. grazing effects across all three grassland types as determined by one-way ANOVA followed by Duncan’s multiple range test. **(c)** Correlations between soil microbial respiration and other variables were quantified using Pearson’s method.

Through the Figure 5 analysis, in the 0–5 cm layer, microbial respiration decreased by 71.3% (*p* < 0.01) in the temperate steppe and 31.8% (*p* < 0.05) in mountain meadow compared to pre-enclosure levels (Figure 5a). At 5–10 cm depth, only the temperate desert showed a marked decrease of 74.7% (*p* < 0.05) in microbial respiration after enclosure, while other grassland types remained statistically unchanged (Figure 5b).

Pearson correlation analysis showed that soil microbial respiration was significantly correlated with NH_4_^+^-N, NO_3_^−^-N, MBC, MBN, and fungal Shannon index in the temperate desert after enclosure (*p* < 0.05 and *p* < 0.01). However, there were significant and extremely significant correlations with BGB, TN, and bacterial-βNTI in the mountain meadow (*p* < 0.05 and *p* < 0.01), but no significant correlations with each index in temperate steppe (Figure 5c).

### 3.5 Key drivers of soil microbial respiration under enclosure

**Figure 6:**
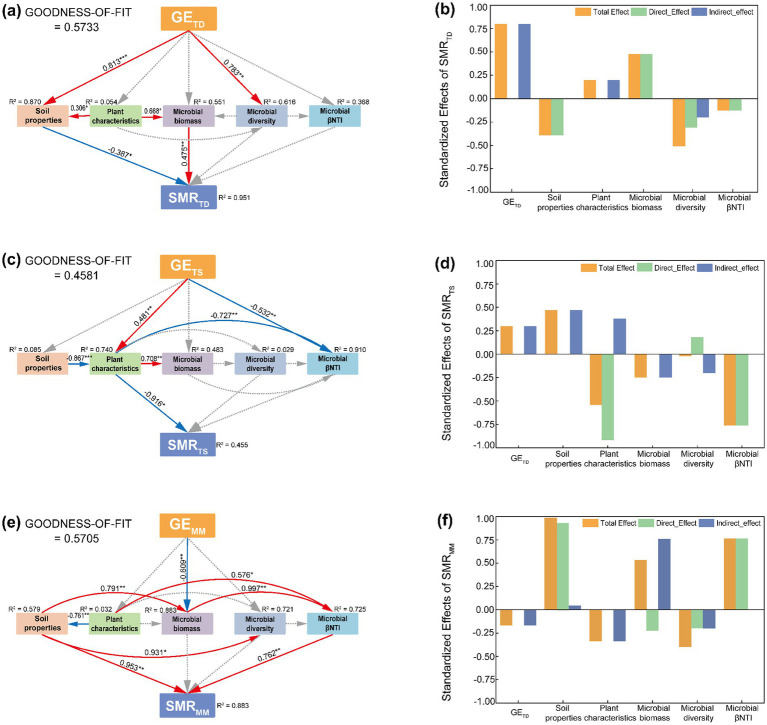
Analysis of soil microbial respiration path in different grassland types under the background of enclosure. **(a)** Temperate desert SEM, **(b)** temperate desert standardized effects of SMR, **(c)** temperate steppe SEM, **(d)** temperate steppe standardized effects of SMR, **(e)** mountain meadow SEM, **(f)** mountain meadow standardized effects of SMR; The numbers on the arrows represent the path coefficient, which are normalized by the mean of each parameter; The red, blue, and dashed gray arrows indicated significant positive correlations (*p* < 0.05), significantly negative correlation (*p* < 0.05) and no significant relationship (*p >* 0.05), respectively; The values near each variable represent the variance interpreted by model (*R*^2^); “*,” “**” and “***” respectively indicated significant (*p* < 0.05), extremely significant (*p* < 0.01) and (*p* < 0.001).

The results of the structural equation modeling revealed that the factors influencing soil microbial respiration varied across grassland types (Figure 6). In the temperate desert, microbial respiration was primarily driven by the direct effects of soil properties and microbial biomass (*p* < 0.05, Figure 6a). Together with microbial diversity and assembly processes, these factors accounted for 95.1% of the variation in microbial respiration (Figure 6a). However, the total effects indicated that grazing exclusion had the strongest influence, suggesting that enclosure measures altered the habitat conditions of the temperate desert grassland, thereby indirectly affecting microbial respiration (Figure 6b). In the temperate steppe, microbial respiration was mainly influenced by plant traits (*p* < 0.05), followed by microbial assembly processes (Figures 6c,d). These factors, along with microbial diversity, collectively explained 45.5% of the variation in microbial respiration (Figure 6c). This pattern contrasted sharply with that observed in the temperate desert. In the mountain meadow, microbial respiration was directly affected by soil properties and microbial assembly processes (*p* < 0.01, Figure 6e), which, together with microbial biomass and diversity, explained 88.3% of the variation in microbial respiration (Figures 6e,f).

## 4 Discussion

### 4.1 Response of microbial community structure and diversity to enclosure

High-throughput sequencing revealed conserved phylum-level dominance patterns in grassland microbial communities, with bacterial assemblages consistently dominated by Actinobacteria, Proteobacteria, Acidobacteria, Bacteroidetes, Verrucomicrobia, and Chloroflexi, while fungal communities were primarily composed of Ascomycota and Basidiomycota (Figures 2a,b). These patterns align with established grassland microbial ecology frameworks, suggesting evolutionary conservation of core taxa with multifunctional adaptations to environmental fluctuations (Wei et al., 2020; Bachar et al., 2010; Chu et al., 2016; Zhang et al., 2013). Specifically, these conserved microbial guilds exhibit high soil niche plasticity, enabled by traits such as xerotolerance, hydric stress resilience, and metabolic versatility across moisture gradients. This adaptive capacity allows them to maintain functional dominance despite grassland-type variations (Li et al., 2019; Ren et al., 2018; Li et al., 2018). Following enclosure implementation, we observed systematic shifts in bacterial functional guilds: oligotrophic groups (Actinobacteria, Acidobacteria) increased in relative abundance, whereas copiotrophic phyla (Bacteroidetes, Firmicutes) declined. This successional transition from fast-growing to slow-growing microbial strategists likely reflects enhanced soil nutrient availability and vegetation diversity post-grazing exclusion (Zhou et al., 2017; Zhang et al., 2018), particularly through increased organic matter inputs that favored resource-competitive Actinobacteria and Acidobacteria. Concurrently, improved soil aeration under enclosure reduced the viability of anaerobic Firmicutes and Bacteroidetes, demonstrating how management-induced physicochemical changes cascade through microbial communities. Fungal communities demonstrated Basidiomycota enrichment across soil profiles, aligning with their lignin-degrading specialization (Lienhard et al., 2014; Ferreira de Araujo et al., 2017) and enclosure-induced accumulation of lignin-rich graminoid litter (He et al., 2019; Li et al., 2019).

Ecosystem-specific *α*-diversity trajectories emerged, with the xeric temperate desert exhibiting the strongest microbial re-assembly after enclosure, while mesic systems remained comparatively stable (Figure 3). New correlative evidence (Supplementary Figure S1) attributes these patterns to management-dependent plant–soil–microbe feedbacks: In the temperate desert, enclosure reversed bacterial linkages-from positive correlations with plant richness/SOC/TP/AP under grazing to negative associations—signaling a resource-driven regime shift where elevated plant inputs intensified competitive exclusion (Supplementary Figures S1a,b) (Cheng et al., 2016); simultaneously, fungi shifted from neutral to positive correlations with BGB/TN/AP, indicating nutrient-mediated functional specialization toward saprotrophic pathways under enrichment (Supplementary Figures S1a,b) (Zhou et al., 2024). In the temperate steppe, bacteria tightened phosphorus coupling (TP/AP, post-enclosure), revealing copiotrophic exploitation of newly available P; fungi decoupled from plant diversity (for Chao1), yet gained positive Shannon links with microbial biomass, demonstrating taxon-specific N-limitation persistence (Supplementary Figures S1c,d). In the mountain meadow, bacterial diversity strengthened ties to plant richness and MBC, confirming edaphic niche creation via litter-root synergy; fungal diversity maintained negative plant correlations, reflecting unwavering competitive exclusion by vegetation (Supplementary Figures S1e,f). Collectively, these data-anchored linkages establish that enclosure duration and ecosystem type jointly determine feedback loop directionality—from resource competition to niche optimization—refining grassland microbial models (Zhang et al., 2018; Zhang and Fu, 2022).

### 4.2 Taxon-specific assembly mechanisms and their environmental drivers under enclosure

Our null model analysis demonstrated that enclosure management induced divergent microbial assembly pathways: bacterial communities shifted toward deterministic selection (βNTI > − 2.0) in arid surface soils, while fungal assembly maintained stochastic dominance (+2.0 < βNTI < −2.0) (Figure 4). Crucially, these patterns were driven by distinct environmental factors: (1) Bacterial deterministic assembly was primarily governed by aboveground biomass (AGB) [*R*^2^ = 0.52, *p* < 0.001; Figure 4A(c)], where enhanced plant inputs intensified niche competition through litter-root synergy, optimizing selective pressures for competitive taxa (Wu et al., 2022; Fierer, 2008; Thuiller et al., 2005; Mason et al., 2011); (2) Fungal stochastic persistence occurred under multi-resource constraints involving belowground biomass (BGB: *R*^2^ = 0.23), soil organic carbon (SOC: *R*^2^ = 0.28), total nitrogen (TN: *R*^2^ = 0.33), and total phosphorus (TP: *R*^2^ = 0.22) [*p* < 0.05; Figure 4B(c)]. Consistent with Dini-Andreote et al. (2015), such heterogeneous resource availability amplifies stochasticity under moderate selection, enabling species-specific physiological adaptations that sustain biodiversity in resource-abundant environments (Chase, 2010).

These results refine classical niche theory (Bahram et al., 2018; Tripathi et al., 2012; Jiao et al., 2022): enclosure promotes deterministic bacterial assembly via plant-derived filters (AGB), aligning with observed competitive exclusion patterns (Section 4.1), while fungi respond to localized resource mosaics (BGB/SOC/TN/TP) through stochastic dynamics that facilitate functional diversification. This establishes taxon-specific environmental drivers as key mediators of microbial stabilization strategies under perturbation.

### 4.3 Response of microbial respiration to enclosure and its driving mechanisms

Our investigation demonstrates that grazing exclusion consistently suppressed soil microbial respiration across three distinct grassland ecosystems (Li et al., 2013), indicating its universal effectiveness in reducing carbon mineralization and potentially enhancing ecosystem carbon sequestration (Figures 5, 6). However, the underlying mechanisms driving this common outcome were strikingly divergent, highlighting the paramount importance of ecosystem-specific context in predicting the response to management interventions.

In the temperate desert grassland, the reduction in respiration was primarily governed by microbial physiological adaptation to nutrient limitation (Figures 6a,b) (Esch et al., 2017). Enclosure improved soil nutrient conditions and promoted microbial biomass accumulation, but it also induced strong stoichiometric imbalances (Supplementary Table S2). This triggered a fundamental metabolic trade-off, where microbes prioritized survival and biomass formation over respiratory activity. Consequently, the community shifted from a fast-growing, high-respiration state to a maintenance-oriented, energy-conserving state, a process consistent with the “microbial carbon pump” concept (Liang et al., 2017; Liang et al., 2019). Here, the direct negative effect of soil properties and microbial nutrient limitation overwhelmed the positive effect of increased biomass, leading to enhanced carbon retention efficiency.

Conversely, in the temperate steppe, the plant community emerged as the central mediator. Enclosure led to a decline in plant diversity, which itself exerted a strong negative effect on microbial respiration, likely through the production of poorer quality litter (Supplementary Table S3; Figures 6c,d) (Wang et al., 2014). Paradoxically, the net respiration decreased, a outcome resolved by a cascade of changes: enclosure fostered a more deterministic microbial assembly process, which selected for a community with a higher carbon use efficiency, thereby enhancing carbon retention—a finding that aligns with the newly identified primacy of microbial CUE in controlling soil carbon storage at a global scale (Figure 4) (Zhang et al., 2022; Tao et al., 2022). This efficiently assembled community effectively counteracted the upward pressure on respiration from both increased plant biomass and soil nutrients. Thus, the microbial response to environmental filtering, rather than plant traits directly, ultimately governed the carbon cycle.

In contrast to both, the mountain meadow exhibited a clear top-down control mechanism (Figures 6e,f) (Aqeel et al., 2023; Reynolds et al., 2003). Enclosure promoted plant growth, which directly and negatively impacted microbial biomass (Supplementary Tables S2, S3). This sharp decline in microbial abundance was the paramount factor suppressing respiration, as evidenced by a strong positive relationship between them (Total effect = 0.53). The reduction in biomass further weakened the microbial assembly process, which itself had a strong positive effect on respiration. This uncoupled the shift toward deterministic assembly from an increase in overall metabolic activity. Despite strong positive direct effects from soil properties, their influence was insufficient to offset the overwhelming suppressive effect of a diminished microbial pool.

Synthesis and Implications: Despite a common outcome, our study reveals three unique pathways through which enclosure moderates the soil carbon cycle: (1) Microbial Metabolic Trade-off in deserts, (2) Microbial Assembly-Mediated Efficiency in steppes, and (3) Plant-Induced Biomass Reduction in meadows. This spectrum of mechanisms—from primarily abiotic (soil nutrients) to biotic (plants and microbes)—underscores that effective ecosystem management must be context-dependent. Our findings move beyond a simple narrative of enclosure increasing carbon stocks and instead illuminate the complex biotic interactions that ultimately determine ecosystem carbon cycling efficiency. Future assessments of grazing exclusion policies should integrate these ecosystem-specific mechanisms to accurately predict their long-term impacts on grassland carbon sequestration.

## 5 Conclusion

Our study demonstrates that grazing enclosure consistently reduces microbial respiration across temperate desert, steppe, and mountain meadow ecosystems, indicating their universal potential to enhance carbon sequestration. However, this common outcome emerges through divergent ecosystem-specific mechanisms. In the temperate desert, reduced respiration was driven by microbial metabolic trade-offs in response to stoichiometric imbalances, enhancing carbon use efficiency. The temperate steppe response was mediated through biotic interactions, where enclosure filtered for a more deterministic microbial assembly process that suppressed respiration despite increased plant biomass. In contrast, the mountain meadow exhibited top-down control where plant-induced suppression of microbial biomass directly drove respiration declines.

While these findings reveal distinct pathways of carbon cycle regulation, the restricted spatial scale necessitates cautious extrapolation. Future research should prioritize temporal analyzes of microbial functional succession, age-dependent responses to enclosure duration, and long-term plant-microbe coevolution monitoring to refine predictive models and evidence-based strategies for ecosystem carbon management.

## Data Availability

The original contributions presented in the study are publicly available. This data can be found here: NCBI BioProject, accession PRJNA1338829.
