# Supplementary material for: From arid deserts to mesic meadows: divergent pathways regulating microbial respiration under grassland enclosure
Source: Front Microbiol. 2025 Oct 27;16:1594877. doi: 10.3389/fmicb.2025.1594877 (PMC12598781; doi:10.3389/fmicb.2025.1594877)
Supplement: Supplementary file 1 [file Supplementary_file_1.doc]

**Appendix Supporting information**

**Appendix 1. Materials and methods:**

**1.Sampling design**

**1.1 Plant sampling and determination**

In July 2021, a field vegetation collection study was conducted within the designated test area. Specifically, three representative transect lines were established in each enclosure treatment area and control area, as depicted in Fig. 1. These transect lines were spaced at intervals of 50 meters apart. Furthermore, three 1 m × 1 m quadrats were systematically placed along each transect line, with approximately 50 meters between each quadrat, totaling 54 sample quadrats.

During the data collection process, the plant species present in each quadrant were carefully recorded. Additionally, the species-specific coverage, height, density, and biomass were meticulously measured. The species coverage (%) was determined using the acupuncture method. The species height (cm) was measured using a standard tape measure. The density (plant·m^-2^) was calculated through direct counting. The above-ground biomass (AGB, g·m^-2^) was determined using the harvest method, and collected samples were transported to the laboratory, where they were initially oven-dried at 105℃ for 30 minutes, followed by a drying period at 80℃ for 24 hours, and then weighed to achieve a constant weight. Finally, the below-ground biomass (BGB), undisturbed soil samples were collected through Block Sampling, where a rectangular soil block (10 cm × 20 cm) was manually excavated with a shovel, maintaining its structural integrity for subsequent analyses, placed in labeled nylon bags with a mesh size of 0.5 mm, and transported to the laboratory for further analysis. The below-ground biomass was subsequently determined through a rigorous process involving washing the samples to remove any residual soil or debris. Following this, the samples were dried at 80℃ for 24 hours to ensure complete moisture removal. After drying, the samples were weighed to obtain an accurate measurement of the below-ground biomass.

## **1.2** **Soil sampling and determination**

Soil samples were collected using the soil borings method within the quadrats where plant community characteristics were assessed (Fig. 1). Stratified sampling was employed, with soil samples taken from the 0-5cm and 5-10cm soil layers. Each sample from each line was evenly mixed separately and placed in a labeled, sealed bag for transportation to the laboratory. A portion of the samples were stored in a refrigerator at 4℃ to maintain their integrity, while the remaining samples were naturally air-dried indoors after removing plant roots, gravel, and other debris. The dried soil samples were then ground and mixed thoroughly. Subsequently, they were sieved through 2 mm, 1 mm, and 0.25 mm screens to ensure a uniform particle size for indoor soil analysis. The processed soil samples were then stored for subsequent laboratory analysis.

Soil pH using the acidometer method (soil and water ratio of 5:1), the soil water content (SWC) was dried at 105℃ and weighed for 24 h, soil bulk density (BD) was adopted by the cutting ring method.

Soil organic carbon (SOC), total nitrogen (TN), total phosphorus (TP), ammonium nitrogen (NH_4_^+^-N), nitrate nitrogen (NO_3_^-^-N) and available phosphorus (AP) contents were successively adopted by dichromate oxidation method, Kjeldahl method, molybdenum antimony colorimetric methods, indophenol blue spectrophotometry, spectrophotometry and Mo-Sb colorimetric method

Microbial biomass carbon (MBC), microbial biomass nitrogen (MBN), and microbial biomass phosphorus (MBP) were fumigated and extracted by chloroform. Fresh soil samples stored at 4℃ were screened by 2 mm and cultured in an incubator at 25℃ for 7~15 days. The cultured soil samples were divided into 6 parts, 3 parts were fumigated with chloroform (3 parts were not fumigated as the control), 40 ml 0.25 mol·L^-1^ K_2_SO_4_ solution was added, shaken and filtered, and the microbial biomass carbon and nitrogen in the extract was determined by TOC analyzer, and microbial biomass phosphorus was determined by spectrophotometer after microwave digestion method.

## **1.3 Data statistics and analysis**

## **1.3.1 Plant diversity**

The Richness index, Shannon-Wiener index, Pielou index and Simpson index were adopted as the plant community diversity indexes.

The calculation formula is below:

Importance value (*P*) *P*=(*H_i_* + *C_i_* + *D_i_* + *B_i_*) / 4 (1)

Richness index (*R*): *R* = *S* (2)

Shannon-Wiener index (*H*): *H* = -∑*P_i_* ln*P_i_* (3)

Pielou index (*E*): *E* = *H*/ln*S*  (4)

Simpson index (*D*): *D* = 1-∑*P_i_^２^* (5)

In the formula, *H_i_* represents the relative height, *C_i_* relative coverage, *D_i_* relative density, *B_i_* relative biomass, *S* is the total species number of the plot; *P_i_* was the important value of the *i* species.

## **1.3.2 Statistical analysis**

First and foremost, Microsoft Excel 2020 was meticulously utilized to preprocess the collected data. Subsequently, IBM SPSS Statistics 25.0 software was utilized to perform independent-sample T tests analysis on the Plant Soil characteristics data collected under both enclosure and grazing conditions. This allowed for a comparative assessment of the factors influencing these characteristics under different management practices. It is worth noting that all data presented in this study are shown as the mean and standard error, providing a robust and reliable representation of the findings. All datasets are original. Plant/soil data will be analyzed in a companion study currently in preparation, with no content overlap between manuscripts.

**Appendix 2. Supplementary table**：

Table S1 Geographical location and basic information of the study sites

| Grassland types | Regions | Geographical position | MAP/mm | MAT/℃ | Elevation(m) | Enclosure years |
| --- | --- | --- | --- | --- | --- | --- |
| Temperate desert | Fukang City | E88.15°，N44.39° | 145 | 5.9 | 514 | 9 |
| Temperate steppe | Wenquan County | E80.55°，N45.01° | 210 | 3.6 | 2338 | 9 |
| Mountain meadow | Qitai County | E89.59°，N43.62° | 420 | 5.5 | 2611 | 9 |

**2.1 Effects of enclosure onsoil physicochemical properties of different grassland types**

Enclosure induced stratified nutrient dynamics across grasslands (Table S2). While pH remained stable (*P*>0.05), 5-10 cm soil bulk density decreased significantly in temperate desert and mountain meadow (*P*<0.05). Mountain meadow exhibited depth-dependent shifts: 0-5 cm SOC and TN decreased (*P*<0.05), contrasting with 5-10 cm SOC and TP increases (*P*<0.05). Temperate desert demonstrated opposing NO_3_^-^-N (-57.2% and -45.4%) and AP (+69.9% and +110.6%) trends between layers (*P*<0.05, Table S2).

Table S2 demonstrates significant changes in MBC content in the 0-5 cm layer of temperate steppe and mountain meadow following enclosure, with a 11.5% increase and 38.2% decrease (*P*<0.05), respectively. In contrast, MBN in the 0-5 cm and 5-10 cm layers of temperate desert showed a decrease-then-increase trend (*P*<0.05), while in the temperate steppe, it followed an opposite pattern (*P*<0.05). Additionally, MBP content significantly decreased by 72.9% and 38.5% in the 0-5 cm layer of temperate desert and mountain meadow, respectively (*P*<0.05).

**Table S2 Effects of enclosure on soil physical and chemical** **properties of different grassland types**

| index | TD _0-5 cm_ | | TD _5-10 CM_ | | TS _0-5 cm_ | | TS _5-10 CM_ | | MM _0-5 cm_ | | MM _5-10 CM_ | |
| --- | --- | --- | --- | --- | --- | --- | --- | --- | --- | --- | --- | --- |
|  | FG | GE | FG | GE | FG | GE | FG | GE | FG | GE | FG | GE |
| pH | 9.59±0.21a | 9.39±0.40a | 9.62±0.30a | 9.67±0.37a | 7.67±0.28a | 7.64±0.14a | 7.64±0.08a | 7.43±0.15a | 6.78±0.18a | 6.98±0.11a | 6.67±0.27a | 6.52±0.18a |
| SWC (%) | 0.02±0.01a | 0.02±0.01a | 0.02±0.00a | 0.02±0.00a | **0.05±0.01a** | **0.03±0.01b** | 0.06±0.02a | 0.06±0.01a | 0.18±0.04a | 0.18±0.03a | **0.17±0.01b** | **0.21±0.01a** |
| BD (g·cm^-3^) | 1.48±0.11a | 1.37±0.10a | **1.53±0.04a** | **1.38±0.06b** | 1.31±0.15a | 1.26±0.13a | 1.27±0.16a | 1.15±0.13a | 0.82±0.09a | 0.74±0.03a | **1.02±0.08a** | **0.84±0.04b** |
| SOC (g·kg^-1^) | 1.22±0.26a | 2.92±1.07a | 0.87±0.29a | 1.27±0.14a | 27.61±1.37a | 23.78±3.38a | 25.46±2.77a | 27±1.68a | **95.75±5.98a** | **74.96±7.57b** | **60.77±0.67b** | **73.26±3.75a** |
| TN (g·kg^-1^) | 0.16±0.07a | 0.27±0.05a | 0.07±0.01a | 0.05±0.01a | 2.31±0.20a | 2.38±0.20a | 2.74±0.48a | 3.17±0.29a | **10±0.33a** | **8.41±0.17b** | 6.46±0.76a | 7.95±0.67a |
| TP (g·kg^-1^) | **0.44±0.01b** | **0.6±0.05a** | **0.44±0.02b** | **0.59±0.03a** | 0.51±0.06a | 0.48±0.04a | 0.53±0.01a | 0.54±0.06a | 0.99±0.04a | 0.93±0.09a | **0.75±0.07b** | **0.91±0.04a** |
| NH_4_^+^-N (mg·kg^-1^) | 4.79±0.73a | 5.72±0.92a | 3.87±0.49a | 3.39±0.40a | 3.91±0.56a | 4.48±0.95a | 4.79±0.68a | 5.47±1.05a | 10.58±0.89a | 10.63±1.67a | 9.98±1.18a | 13.01±1.63a |
| NO_3_^-^-N (mg·kg^-1^) | **48.63±4.88a** | **20.83±2.62b** | **30.84±2.53a** | **16.83±1.45b** | 5.84±0.45a | 6.35±1.84a | 6.07±1.11a | 4.33±0.55a | 10.55±1.48a | 6.91±2.18a | 11.49±1.15a | 10.17±2.11a |
| AP (mg·kg^-1^) | **11.97±2.49b** | **20.35±2.12a** | **8.83±1.19b** | **18.59±2.51a** | 17.65±3.78a | 18.17±2.49a | 17.28±0.81a | 15.92±1.24a | **34.64±3.89a** | **27.67±0.09b** | 26.34±2.18a | 29.15±2.47a |
| MBC (mg·kg^-1^) | 266.21±22.31a | 270.47±40.94a | 82.06±36.78a | 149.69±23.19a | **610.16±13.74b** | **680.21±39.47a** | 516.06±42.72a | 467.52±47.14a | **1469.74±152.24a** | **909.04±37.56b** | 1040.12±127.19a | 957.66±150.77a |
| MBN (mg·kg^-1^) | **9.8±2.00a** | **2.3±0.92b** | **13.43±2.29b** | **25.42±6.55a** | **19.55±4.74b** | **44.5±10.74a** | **23.19±2.97a** | **3.49±0.55b** | **145.63±16.76a** | **81.45±18.26b** | 81.33±11.05a | 61.57±10.81a |
| MBP (mg·kg^-1^) | **31.84±2.09a** | **8.62±3.50b** | 4.3±2.80a | 5.17±1.91a | 21.25±4.95a | 30.68±5.77a | 24.08±12.19a | 18.33±3.40a | **100.58±9.30a** | **61.83±3.39b** | 60.07±14.96a | 68.14±13.16a |

Note: pH, pH degree of acid or alkali; SWC, soil water content; BD, bulk density; SOC, soil organic carbon; TN, total nitrogen; TP, total phosphorus; AP, available phosphorus; NO_3_^-^-N, nitrate nitrogen; NH_4_^+^-N, ammonium nitrogen; MBC, microbial biomass carbon; MBN, microbial biomass nitrogen; MBP, microbial biomass phosphorus.

Different lowercase letters indicate significant differences among various treatments of the same grassland type, (*P*<0.05), while the same lowercase letters indicated no significant differences among various treatments of the same grassland type (*P*>0.05); Bold data indicated significant effects (*P*<0.05), TD、TS、MM、GE and FG respectively represent temperate desert、temperate steppe、mountain meadow、grazing exclusion plots and freely grazing plots; the same below.

**2.2 Effects of enclosure on quantitative characteristics and diversity of the plant community of different grassland types**

Enclosure elicited biome-specific vegetation changes (Table S3). Temperate steppe (TS) and mountain meadow (MM) showed significant increases in plant height (89.5%, 34.6%) and coverage (41.8%, 28.5%) versus grazed areas (P<0.05), contrasting with stable parameters in temperate desert (TD). Universal density declines occurred across ecosystems (TD:59.3%, TS:45.6%, MM:24.8%; P<0.05), while aboveground biomass increased specifically in TS (137.5%) and MM (47.3%) (P<0.05). Stratified analysis of belowground biomass revealed contrasting depth-dependent responses to enclosure (Table S3). In surface soils (0-5 cm), TD exhibited a significant 18.2% increase (P<0.05), whereas TS and MM showed reductions of 12.5% and 9.8%, respectively. Conversely, the subsurface layer (5-10 cm) demonstrated selective enhancement with MM displaying a 140.3% surge (P<0.05), while TD and TS remained unaffected.

Enclosure-driven diversity alterations displayed ecosystem specificity (Table S3). MM demonstrated positive responses with 21.6% and 8.2% increases in species richness and Shannon-Wiener index (P<0.05). In contrast, TD and TS exhibited significant reductions across multiple indices:TD: Richness (-30.4%), Shannon (-42.4%), Simpson (-38.1%); TS: Richness (-40.3%), Shannon (-32.6%), Simpson (-21.1%), (all P<0.05).

Table S3 Effects of enclosure on plant community structure and diversity of different grassland types

| Plant index | TD | | TS | | MM | |
| --- | --- | --- | --- | --- | --- | --- |
|  | FG | GE | FG | GE | FG | GE |
| Height (cm) | 78.81±16.63a | 72.57±19.03a | **5.57±0.61b** | **10.56±2.77a** | **25.52±9.96b** | **34.34±3.60a** |
| Cover (%) | **6.1±3.28a** | **2.23±1.84b** | **37.73±2.96b** | **53.5±4.87a** | **109.5±16.24b** | **140.67±24.74a** |
| Density (plant·m^-2^) | **4.2±2.62a** | **1.71±1.66b** | **117.11±36.79a** | **63.67±15.00b** | **229.33±57.26a** | **172.44±31.55b** |
| Above-ground biomass (g·m^-2^) | **23.11±13.41a** | **12.32±5.44b** | **32.06±12.60b** | **76.13±31.76a** | **69.03±37.47b** | **101.7±20.43a** |
| 0-5cm BGB (g·m^-2^) | **42.1±50b** | **65.1±11.03a** | **313.6±32.11a** | **180.27±23.15b** | 966.77±237.58a | 865.82±43.01a |
| 5-10cm BGB (g·m^-2^) | 23.58±10.63a | 6.84±1.53a | 194.68±58.97a | 117.38±11.85a | **258.83±36.10b** | **621.93±66.83a** |
| Richness index | **2.56±0.73a** | **1.78±0.67b** | **8.56±0.88a** | **5.11±1.27b** | **11.33±2.06b** | **13.78±1.09a** |
| Shannon-Wiener index | **0.86±0.25a** | **0.49±0.39b** | **1.8±0.14a** | **1.21±0.24b** | **2.2±0.22b** | **2.38±0.09a** |
| Pielou index | 0.95±0.04a | 0.65±0.49a | **0.84±0.05a** | **0.77±0.08b** | 0.91±0.04a | 0.91±0.03a |
| Simpson index | **0.55±0.10a** | **0.34±0.26b** | **0.77±0.05a** | **0.61±0.09b** | 0.86±0.04a | 0.89±0.02a |

Note: BGB, below-ground biomass; Bold data indicated significant effects (*P*<0.05), TD、TS、MM、GE and FG respectively represent temperate desert、temperate steppe、mountain meadow、grazing exclusion plots and freely grazing plots.

**2.3 Spearman correlation between plant, soil and microbial properties**

Across grassland types, grazing exclusion induced shifts in the relationships between microbial community diversity and plant-soil properties, as indicated by Spearman correlations (Fig. S1). Fig. a: Temperate desert freely grazing plots (FG): Bacterial diversity: Bacterial Chao1 and Shannon indices showed positive correlations with AGB, SWC, SOC, TP, and AP, and negative correlations with TN, NH₄⁺-N, and NO₃⁻-N; Fungal diversity: Fungal Chao1 and Shannon indices were positively correlated with plant Richness and Shannon-Wiener indices.

Fig. b: Temperate desert grazing exclusion plots (GE): Bacterial diversity: Bacterial Chao1 and Shannon indices exhibited negative correlations with plant Richness, Shannon-Wiener indices, and SOC; Fungal diversity: Fungal Chao1 and Shannon indices showed positive correlations with BGB, TN, AP, NH₄⁺-N, MBC, and MBP.

Fig. c: Temperate steppe freely grazing plots (FG): Bacterial diversity: Bacterial Chao1 and Shannon indices were positively correlated with plant Richness, SOC, TP, NH₄⁺-N, MBC, MBN, and MBP; Fungal diversity: Fungal Chao1 was positively correlated with AGB, BGB, and SOC, and negatively correlated with TN, TP, AP, NH₄⁺-N, and NO₃⁻-N.

Fig. d: Temperate steppe grazing exclusion plots (GE): Bacterial diversity: Bacterial Chao1 and Shannon indices were positively correlated with SOC, TN, TP, and AP; Fungal diversity: Fungal Chao1 was negatively correlated with plant Richness, Shannon-Wiener indices, AGB, SOC, TN, and TP, and positively correlated with MBC, MBN, and MBP. Fungal Shannon index showed positive correlations with plant Richness, Shannon-Wiener indices, AGB, MBC, and MBN.

Fig. e: Mountain meadow freely grazing plots (FG): Bacterial diversity: Bacterial Chao1 and Shannon indices were positively correlated with BGB, SWC, SOC, TN, TP, AP, MBC, and MBP; Fungal diversity: Fungal Chao1 and Shannon indices showed negative correlations with plant Richness and Shannon-Wiener indices, and AGB. Fungal Chao1 was positively correlated with SOC, TN, TP, and AP, while Fungal Shannon index was negatively correlated with these variables.

Fig. f: Mountain meadow grazing exclusion plots (GE): Bacterial diversity: Bacterial Chao1 and Shannon indices were positively correlated with plant Richness, Shannon-Wiener indices, AGB, TP, and MBC.


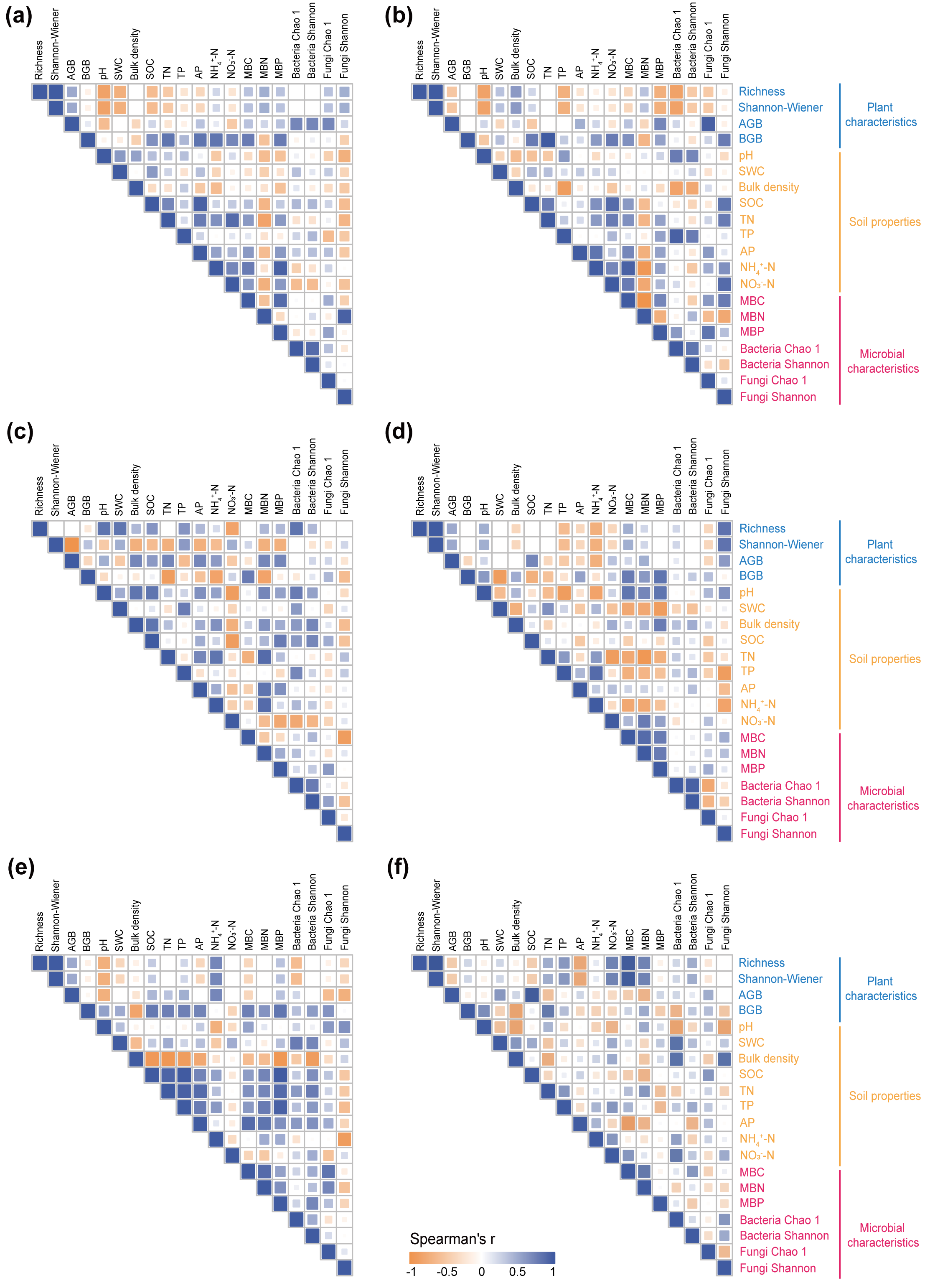


Note: Relationships between variables were analyzed using Spearman correlation; Fig. a: temperate desert freely grazing plots, Fig. b: temperate desert grazing exclusion plots, Fig. c: temperate steppe freely grazing plots, Fig. d: temperate steppe grazing exclusion plots, Fig. e: mountain meadow freely grazing plots, FG; Fig. f: mountain meadow grazing exclusion plots, GE; AGB (above-ground biomass), BGB (below-ground biomass), pH (pondus hydrogenii), SWC (soil water content), SOC (soil organic carbon), TN (total nitrogen), TP (total phosphorus), AP (available phosphorus), NH_4_^+^-N (ammonium nitrogen), NO_3_^-^-N (nitrate nitrogen), MBC (microbial biomass carbon), MBN (microbial biomass nitrogen), MBP (microbial biomass phosphorus) .

Fig. S1 Spearman correlation between plant, soil and microbial properties
